# Saliva: Both a Threat and an Opportunity in Covid-19 Pandemic

**DOI:** 10.12669/pjms.37.4.4263

**Published:** 2021

**Authors:** Ismail Serhat Sadikoglu, Mehmet Gagari Caymaz

**Affiliations:** 1 Dr. Ismail Serhat Sadikoglu, DDS, PhD. Department of Restorative Dentistry, Near East University Faculty of Dentistry, Nicosia, Mersin10, Turkey; 2 Dr. Mehmet Gagari Caymaz, DDS, PhD. Department of Oral and Maxillofacial Surgery, Near East University Faculty of Dentistry, Nicosia, Mersin10, Turkey

**Keywords:** Saliva, Diagnostics, SARS-CoV-2, Covid-19

## Abstract

Covid-19 pandemic, continues all over the world with the increasing number of confirmed cases and performed tests day by day. It has been shown that collecting nasopharyngeal samples, as the most commonly prefered method to perform RT-PCR, has disadvantages like causing discomfort and bleeding in patients. Sample collecting procedure also renders healthcare professionals by exposing them to the risk of transmission of the virus related to the direct contact with patients. These disadvantages make this procedure undesirable for the researchers and forces them to search for an alternative technique. At this point, saliva appears as an opportunity, regarding its high viral load. On the other hand, this high viral load poses a threat, especially for professions such as dental practitioners, with too much aerosol exposure. Since dentistry is a branch of health that constantly needs direct operations, it is necessary to be protected from the virus as much as possible while caring for the patient. A literature review was done using electronic databases “PubMed,” “Google Scholar,” and “Cochrane Database,” on January 2021. Studies have proposed many different preventive measures in this regard. Therefore, the purpose of this review is to draw attention to the saliva by bringing together the recent research and also to provide information and a perspective to dental clinicians about both prevention and a potential diagnostic technique.

## INTRODUCTION

New coronavirus disease (COVID-19) has spread to more than 200 countries globally. Worldwide, a total of 105,658,476 confirmed cases of COVID-19, of which 2,309,370 deaths were reported to WHO till 08 February 2021.^1^ It was previously known that Severe Acute Respiratory Syndrome-Coronavirus (SARS-CoV) and Middle East Respiratory Syndrome- Coronavirus (MERS-CoV) affect humans. Outbreaks of respiratory diseases caused by these viruses, which caused ~ 800 deaths during 2002 (SARS) and 860 deaths during 2012 (MERS-CoV) appear to have occurred in animals before passing on to other hosts, such as humans.^2^ While MERS-CoV was transmitted from camels to humans, SARS-CoV passed from cats to humans. Emerging nearly eight years after the MERS-CoV outbreak SARS CoV-2 appears to be a zoonotic pathogen originating from bats, and the first reports of cases come from Wuhan, China’s Hubei Province, and show an animal-human spread from a live animal market. The virus then spread outside of Hubei and then to the rest of the world through human transmission.^3^ On January 30, 2020, the World Health Organization (WHO) declared COVID-19 a Public Health Emergency of International Concern (PHEIC).^4^ Surprisingly, a devastating number of new cases were reported globally in March, above which WHO declared the coronavirus disease as a pandemic on March 11, 2020.^5^ Detection of SARS-CoV-2 is important to avoid the spread of the infection. Since last year’s outbreak, the whole genome / RNA sequencing has been used by the research groups and the viral aetiology of COVID-19, with the genetic sequence showing ~ 80% similarity to the genome of severe acute respiratory syndrome virus (SARS-CoV), has been determined. The novel coronavirus was therefore named as SARS-CoV-2.^6^

Coronaviruses are enveloped RNA viruses which are presenting the classic structure with “spike protein” in the membrane envelope. The interplay between this spike protein and angiotensin converting enzyme 2 (ACE 2) receptors are in charge of the virus entrance into the cells. The dispersion of ACE-2 receptors in various parts of the body may show us the possible routes of infection.^7^ The membrane that attaches to ACE 2 can be found in several tissue cells, including mucosal tissues, gingival tissues, tongue epithelial cells, and salivary glands.^8^ High amount of SARS-CoV-2 viral load in saliva has been reported too and has been propund to exist even in periodontal pockets.^9^ These mentioned statements have also been noted in previous studies suggesting that virus spread may be closely linked to saliva interactions.^10^ It has also been shown that saliva interactions make the oral tissues a potential reservoir for SARS-CoV-2 contamination due to aerosol generation during dental treatments also during coughing, sneezing and speech.^9^,^11^

Currently, direct contact and/or spreading of air droplets are the most likely routes of transmission. This is reinforced by findings that SARS-CoV-2 can be isolated in aerosol (<5μm) for at least three hours.^12^ Thus saliva has an important role in transmission with its high viral load. In our previous study, we came to the conclusion that dentists lacked information about Covid-19 and that they were deficient in protection against aerosol occurring during dental procedures.^13^ Therefore, our aim in writing this review was to draw attention to the areas where saliva is an opportunity and a threat, in the light of the latest research published during this pandemic period. Apart from that objective, in terms of clinical applicability, the purpose of this review is to enable the dental team to understand the importance of protection from high viral load aerosol and act accordingly. In addition, to explain the effect of saliva as a diagnostic method based on evidence and to show that it can be an alternative method compared to the oro-naso-pharyngeal swap method, which is an uncomfortable technique for both patients and healthcare workers.

### Sources and Data

A literature search was done using electronic databases “PubMed,” “Google Scholar,” and “Cochrane Database,” on January 2021 and these were used to identify the articles in English language that cover the relevant objectives. Multiple search keywords were used, such as saliva and covid-19, covid-19 diagnosis, and dentistry and covid-19.

### Saliva: A Friend or An Enemy

Saliva is a special body liquid produced by the major and minor salivary glands. This special liquid is formed mostly of water (94-99%), with 0.5% organic substances and 0.2% inorganic substances. Saliva has a crucial role in cleaning, digesting food, protecting the oral cavity, lubricating the oral mucosa and fulfillment of the balance in the oral cavity. A healthy adult generally produces about 600 ml of saliva each day. Apart from secretion from salivary glands, saliva also carries white blood cells, exfoliated epithelial cells, serum elements and oral microorganisms with their metabolites. More than 700 microbial species which are associated with several diseases have been identified in saliva. It offers a suitable place for the colonization and growth of oral microorganisms. Additionally, it prevents the overgrowth of specific pathogens to protect the natural balance of the oral cavity. Saliva can also act as a keeper and block the passage of pathogens to the respiratory tract and digestive system.^9^

There are at least three separate ways for SARS-CoV-2 to occur in saliva: (1) Infection of the salivary glands release particles from the salivary ducts into saliva; (2) SARS-CoV-2 in the blood can reach the oral cavity through gingival fluid; and (3) Saliva reaches the mouth together with the droplets of the virus in the upper and lower respiratory tract.^14^

The most widely adopted way of SARS-CoV-2 spread is direct contact with respiratory droplets. The use of dental instruments such as high speed and low speed turbines or ultrasonic cleaners in daily dental practice causes splashes of saliva and blood droplets.^15^ The droplets can travel various distances depending on their size. The bigger droplets tend to collapse quickly. Droplets smaller than 5μm can evaporate and become aerosol. Therefore, it lends to airborne transmission of infections.^16^

After ultrasonic cleaning, contamination of the inner surfaces of clinical clothes and masks with saliva has been reported. Even though surgical masks defend the mucous membrane in the oral cavity and nose from splashing, they can not ensure full protection against infections.^15^

Our knowledge about the constancy of the SARS-CoV-2 in saliva droplets or aerosols is very limited. A study showed that the SARS-CoV-2 genome was discoverable in 66.7% of air samples taken from corridors outside patient rooms in addition to air samples taken from the rooms of the patients.^16^ Van Doremalen et al.^12^ looked at the stability of the SARS-CoV-2 and SARS-CoV-1 in aerosols. They stated that the SARS-CoV-2 remained stable in aerosols over the 3-hour test period just like SARS-CoV-1.

All these observations demonstrate an elevated risk of airborne transmission of the SARS-CoV-2 to dental practitioners. Apart from direct spread there is also an indirect way too. Liu et al.^17^ examined thirty-five aerosol samples taken from patient spaces and healthcare personnel spaces at two medical centers for COVID-19 virus by digital polymerase chain reaction. They found that the droplets gathered on the surfaces of workstations were positive for the virus. Van Doremalen et al.^12^ investigated the stability in a more controlled medium and found that lab-fabricated COVID-19 droplets remained stable for three days on various surfaces. In dental clinics, dentists with hands smeared with saliva that continuously touch dental tools and return to the patient’s mouth during the treatment process, can rapidly elevate the ratio of contaminated planes. This wrong application will increase the risk of contamination for all dental team who can reach the clinic and also increase the risk of cross-contamination between patients over a prolonged period of time.^18^

### Protection Suggestions

Researchers suggest different protection measurements to avoid Covid-19 in dental practice. These suggestions mentioned the importance of personal protective equipment (PPE) for dental professionals. Also includes using mouth rinses before dental treatments and using rubber-dam during dental approaches.

### The importance of personal protective equipment

The major way of spread is the airborne droplet in dental practices. Therefore, during the COVID-19 pandemic, all the protection barrier equipment is a priority for all dental professionals. Medical hand gloves, medical face mask, protective eyewear, medical cap, face shield, and special protective suits are examples for PPE. Medical Goggles provide the most trustworthy eye protection from aerosols, but also the face shields are stated as an alternative as an eye contamination protection measurement. Studies report that N95 respirator masks or FFP3 respirator are recommended in addition to room ventilation to avoid airborne transmission.^11^,^19^

### Using mouth rinse before dental procedures

In their recent review, Vergara-Buenaventura and Castro-Ruiz^20^ recommend mouth rinse with different active agents before dental treatments to reduce the COVID-19 viral load prior dental applications and reduce the risk of cross-infection when taking care of the patients throughout the pandemic. Hydrogen peroxide, Povidone-iodine, Chlorhexidine and Cetylpyridinium chloride are the suggested antiseptic solutions to use. Different studies have suggested the use of these antiseptic substances in different concentrations which are summarized in Table-I. All researchers include in the Table-I recommends rinsing for 30 seconds in the mouth and 30 seconds in the back of the throat respectively with the suggested antiseptic solution (Table-I).^21^-^25^

**Table-I:** Suggested antiseptic solutions prior to dental applications and their ratios.

	Active agent	Suggested solution ratios	Suggested volume of the solution
American Dental Association^[35]^	Hydrogen Peroxide	1.5%	15 ml
American Dental Association^[35]^	Povidone-iodine	0.2%	9 ml
Mady et al.^[36]^ Challacombe et al.^[37]^		0.4% 0.5%	
Yoon et al.^[39]^	Chlorhexidine	0.12%	15 ml

### Using rubber-dam during dental treatments

The dental rubber dam is an ideal equipment that is used for isolation in the dental practice, especially during endodontic and adhesive procedures. Rubber-dam helps to reach a proper cross-infection control for both the dental professionals and the patients. The dam provides an excellent isolation from the oral cavity, thus aerosol is not mixed with the saliva of the patient. Which has a high viral load as mentioned before in this review. Several studies report that using the rubber dam is an excellent barrier to the potential spread of the infectious disease in the dental practice.^26^ Dentistry is in a high contamination risk profession group. Therefore, all dental practitioners should avoid the transmission by using these protective measurements in their daily routine. Apart from the virus spreading threat, Saliva has a very important role as a potential diagnostic tool.

### Covid-19 Detection Techniques: A Promising Oppurtunity

The real-time polymerase chain reaction (RT-PCR) with samples taken from nasopharyngeal swabs is accepted as a gold standard currently. A nasal mid-turbinate swab by a healthcare team or self-collection with a conical swab is also accepted as an alternative. However, various studies stated that the samples taken by this method have low sensitivity for detecting SARS-CoV-2.^27^ In addition, close contact during collection of samples elevates the risk of healthcare staff contamination.^28^

Generally, samples taken from the upper respiratory tract as nasopharyngeal or oropharyngeal swabs. Additionally, for patients presenting with pneumonia, they can also be collected from the lower respiratory tract as sputum or endo-tracheal aspirate.^29^ As stated in several papers, nasopharyngeal and oropharyngeal sample collection caused discomfort and bleeding, especially in individuals with thrombocytopenia. This collection method also subject healthcare professionals to the risk of transmission of the virus during sample collection. Thus these causes making it an undesirable method.^29^,^30^

Apart from physical discomforts, pychological effect of repetitive diagnostic and treatment procedures (like oropharyngeal swap, naso pharyngeal swap etc.) should be considered too. Zaidi and Jawaid were reported that moral trauma caused by the COVID-19 has left indelible scars on the human psyche.^31^ Thus, finding another safe to use technique is crucial.

### Comparing Naso-Oro-Pharyngeal Swab Samples With Saliva

Vaz et al.^32^ showed that the use of self-collected saliva specimens is an easy, appropriate, and cheap alternative to traditional nasopharyngeal swab-based tests. These results could lend the wider use of molecular testing to handle the COVID-19 outbreak, particularly in resource-limited settings.

Azzi et al.^33^ declared that COVID-19 virus was detected in the saliva swab in all infected individuals on the first day and after 4 days in their recent research. All individuals were classified as severe or very severe COVID-19. Drooling technique was used to collect saliva. In addition to this, To et al.^30^ mentioned that SARS-CoV-2 RNA was discovered in the saliva of all patients except three individuals who were intubated. They ran a cohort study including 23 individuals with laboratory-confirmed COVID-19. Ten patients classified severe COVID-19, all requiring oxygen support, and remaining 13 patients classified as mild COVID-19.

Saliva collection procedure or nasopharyngeal swabs applied to 44 hospitalized COVID-19 patients by healthcare professionals and 121 samples obtained in the research by Wylli et al.^27^ Positive results were detected in 39 and 46 samples of saliva and nasopharyngeal swabs, respectively. Eight clients had positive saliva and negative nasopharyngeal swab specimens, three cases had negative saliva and positive nasopharyngeal specimens. The effectiveness of saliva as a sample of the COVID-19 virus monitoring test was also investigated. Totally, 39/622 patients had positive in nasopharyngeal swabs and 33 of 39 individuals had SARS-CoV-2 in saliva.^34^ These studies proved that SARS-CoV-2 is detected from saliva as effectively as in nasopharyngeal swabs.

However, in another study, SARS-CoV-2 was discovered in only four saliva samples of 31 individuals whose oropharyngeal swabs were positive.^35^ To prevent contamination with other secretions from the respiratory tract, saliva was collected from the opening of the gland duct of the cleaned oral cavity. The low positive rate of this study may be because of the collection technique.

Studies have shown that saliva specimens have higher viral load or lower cycle threshold against nasopharyngeal swab specimens.^27^,^34^ The sensitivity of saliva samples in detecting COVID-19 virus was also observed. On the same days when pharyngeal or respiratory swabs transformed, the saliva results of two patients were positive.^33^ Less versatility was attend in the SARS-CoV-2 test repeated with a saliva sample.^27^ These results demonstrated that saliva can be a trustworthy specimen for SARS-CoV-2 detection. Laboratory and clinical reviews reported that PCR kits for saliva samples have already been approved by the US Food and Drug Administration (FDA) and are available worldwide.

Under the limitations of this review, most of the researchers stated no significant difference in viral load between nasopharyngeal and saliva samples. These studies concluded that saliva is a non-invasive sample type for the diagnosis of COVID-19 virus and viral load monitoring.^29^,^30^,^33^,^34^,^36^ Studies also declared a high agreement between saliva and nasopharyngeal swab samples when tested by an automated multiplex molecular tests approved for point-of-care testing.^37^,^38^

Several groups have also studied the potential of saliva as a clinical sample for COVID-19 virus detection. In this regard, a study of 12 patients confirmed by PCR detection of viral RNA using nasopharyngeal or sputum samples found that coughing saliva from 11 patients was positive for SARS-CoV-2. Importantly, no viral RNA was detected in saliva samples collected from 33 patients whose nasopharyngeal samples tested negative for SARS-CoV-2. Consistent results were obtained by the same group examining a different set of patients, showing that SARS-CoV-2 was detectable in self-collected saliva of 20 out of 23 confirmed patients.^29^

Another approach to collecting saliva samples is through oral swabs that are easily administered even for non-professionals. In two studies examining saliva samples collected with oral swabs, 15 (50%) of 39 patients and 25 (100%) of 25 patients, respectively, tested positive for SARS-CoV-2.^29^,^36^ While it’s too early to draw any conclusions, these studies indeed imply that saliva collected by coughing or oral swabs is a legitimate clinical sample for SARS-CoV-2 detection.

### Covid-19 Detection With Saliva

The approved saliva-based COVID-19 test kit is built on the current TaqPath SARS-CoV Test developed by the Rutgers Clinical Genomics Laboratory to qualitatively identify RNA from the virus. This test uses primers and probes for respiratory, nasopharyngeal and oropharyngeal samples, validated by the Emergency Use Authority (EUA). Collection protocols and nucleic acid extraction buffers are changed to ensure that the saliva sample is tested. Saliva samples can be transported and stored at ambient temperature but must be processed within 48 hours of collection. The recommended system for RNA extraction is Chemagic Viral DNA / RNA 300 Kit H96 and PerkinElmer ChemagicTM 360. The RT-PCR can be performed using Applied Biosystems TaqPath Combo Kit on the ThermoFisher Applied Biosystems QuantStudio 5 Real-Time PCR System or the Applied Biosystems ViiA7 Real-Time PCR System.^39^

### How should we perform the saliva testing?

The saliva collection method and types of collection devices are critical issues in this detection technique. There are three main types of saliva which includes whole saliva, parotid gland and minor gland samples and the collection method of each type varies accordingly. When the aim of sampling is to detect respiratory viruses by molecular analysis, it is beneficial to collect all types of saliva from suspected patients.^38^ Thus, patients should be informed to drain saliva into a sterile bottle. Saliva volume should be between 0.5 and 1 ml. 2 ml of viral transport medium (VTM) should then be added to the bottle.^40^ Following applications will be performed according to the instructions of the relevant RT-PCR technique in the microbiology laboratory. The poor compliance rate of saliva with nasopharyngeal specimens reported in the research by Chen et al.^35^ and can be clarified by the variation in the sampling method. The research by Chen et al. also stated the detection rate of COVID-19 virus in pristine salivary fluid excreted from the entrance of the gland ducts. On the other hand, in different researches, patients were asked to cough saliva from their throats into sterile bottles, and thus saliva specimens were generally sputum from the lower respiratory tract.^29^,^36^ Therefore, the instructions should understandably clarify the appropriate procedure to patients to elevate the sensitivity of saliva tests on the way to detect suspected COVID-19 individuals.

## CONCLUSION

In daily dental practice, high-speed water-cooled turbines are used frequently. Therefore a high amount of aerosol is created. The virus in this aerosol remained stable for three hours on air and 72 hours on stainless steel or plastic surfaces. Dentist should always protect himself with protective clothing. Additionally, the risk of contamination can be reduced by protective measures such as gargling with various antiseptic solutions before the procedure and performing dental procedures under rubber dam isolation. The high viral load in saliva is both a threat and an opportunity for humanity. Under the limitation to the results of the studies reported in this review, the use of saliva in the diagnosis of Covid-19 is a cost-effective, reliable and easy-to-apply technique compared to other disturbing detection forms. Further salivary studies should be encouraged to reduce the research gap in this area.

### Authors Contribution:

**ISS:** conceived, designed and write the manuscript **MGC:** did data collection review, editing of manuscript and final approval of manuscript
